# Supplementary material for: Patterns of Intron Gain and Loss in Fungi
Source: PLoS Biol. 2004 Nov 30;2(12):e422. doi: 10.1371/journal.pbio.0020422 (PMC532390; doi:10.1371/journal.pbio.0020422)
Supplement: Table S1 — Also available at http://genes.mit.edu/NielsenEtAl/. (4.3 MB ZIP). [file pbio.0020422.st001.zip › NielsenEtAl/html/354.html]

AN4864.1.NCU03317.1.MG09310.1.FG05607.1


```
 CLUSTAL W (1.82) Multiple Sequence Alignments - Introns Inserted


Sequence 1: NCU03317.1	571 aa
Sequence 2: MG09310.1	599 aa
Sequence 3: FG05607.1	597 aa
Sequence 4: AN4864.1	591 aa
Alignment Length: 612 aa
Number Identitical Residues: 275 aa
Alignment Score (without introns) 14771


MG09310.1 	MAGQASPSPRKPRARKAKKIVDNDQEQTVLAVRKNEALVRVPTFPLAAFLWPARGSPTQW
NCU03317.1	--------------------------------MEREALVRIPSFPLAAFLWPARSSVSQW
FG05607.1 	MAG-PSPSPHKARRKTPKKPGNGSGTDVKERTPRAEALVRAPAFPLAAFLWPARTSMSQW
AN4864.1  	---MAPSVPSSYRPRKKRKSAALFAGSNNPLTIDAGEGKAAPAFPLVSFLWGARAGVSQW
          	    ... . .    .  .       .              *:***.:*** ** . :**

MG09310.1 	ETLALTLMIVGVFRWAAGLWGYSG1FHKPPLFGDYEAQRHWMEVTTQLPISQWYFHDLQW
NCU03317.1	ELLPLILMAVGLFRWAAGLWGYSG1FEKPPLFGDYEAQRHWMEVTTHLPISQWYFHDLEW
FG05607.1 	EVLPVILMVVGFFRWAAGLWGYSG1FHKPPMFGDYEAQRHWMEVTTQLPISQWYFHDLQW
AN4864.1  	LVLPLILMTVGLFRWAVSLWGYSG1FNTPPMYGDFEAQRHWMEITIHLPLSKWYTYDLQY
          	  *.: ** **.****..****** *..**::**:********:* :**:*:** :**::

MG09310.1 	WGLDYPPLTAYHSWLLGKI~GSLINPAWFALFSSRGSDDPVLKIFMRATVLVSEYLIYVP
NCU03317.1	WGLDYPPLTAYHSWICGKI~GSLIDPAWFALYDSRGSHDPTLKIFMRATVLVSEYLIYIP
FG05607.1 	WGLDYPPLTAYHSWVLGKL~GSYIDPSWFALFTSRGNQDPDLKIFMRATVIVSEYLIYIP
AN4864.1  	WGLDYPPLTAYHSWLLGKI2GSVFDPTLFALDDSRGIEGSLLKVFMRATVVVSEYLVYIP
          	**************: **: ** ::*: ***  *** ... **:******:*****:*:*

MG09310.1 	ATVVFVRRFGRLGGVDRWSSSVALVAILMQPGLILIDHVHFQYNTVMLGFVLASMSSMLA
NCU03317.1	AAVIFVRRFSRLSGVPAWTSSVALVAILMQPATILIDHVHFQYNTVMLGFVLASMSSMLA
FG05607.1 	AAIVFVRRYSRLQGVTNWTAWLALVAILMQPATILIDHVHFQYNTVMLGFVMASMSSMLA
AN4864.1  	AIVTFLRRYTRMQAVPVWSSSIALSAILLQPATILIDHGHFQYNTVMLGLFVASLDAIMA
          	* : *:**: *: .*  *:: :** ***:**. ***** **********:.:**:.:::*

MG09310.1 	GRLLWASVFFVAALGFKQMALYYAFSIFAYLLGACVF-----PKLNLPRLLAIAAVTAIS
NCU03317.1	GRNFWACFFFVMALGFKQMALYYAFSVFAYLLGVCVFDHTNIPRFNIPKFIGIALATVVS
FG05607.1 	ERYKWAAVFFVGALGFKQMALYYAFSVFSYLLGRCVF-----PRIDLTRLFGIALVTAVS
AN4864.1  	GRMLWACIFFVGALGFKQMALYYAPVMFAFLLGICIF-----PRIRLVRLFCIALVTIAS
          	 *  **..*** ************  :*::*** *:*     *:: : ::: ** .*  *

MG09310.1 	FAILVLPLIIGALYDAQRGIDSRPDLDGPPPPLPIFGFLSEYLDTDAVYYPVVEQMIQLI
NCU03317.1	FAILLLPIVAGTLSEASRGITAHP--GGAHPPLPLFADLAKHLNTEAFYYPVIEQLVQMI
FG05607.1 	FAILILPLILGTFYDKTQGIDAHGG-SKDSPSLPLLPQLVNILDTDAFYWPYVEQMVQLV
AN4864.1  	FTALLLPLLLGATSTEAG--------KQPVPEPPLLQAFPVNLDHGSSLYLILFQLTQIV
          	*: *:**:: *:                  *  *::  :   *:  :  :  : *: *::

MG09310.1 	HRVFPFARGLFEDKVANFWCAINVVFKLRKYPAALLQQAALGATLAAIVPPNLILFLRPR
NCU03317.1	HRVFPFARGLFEDKVANFWCAMNVVIKLKQYPAELLQKGALVATLAAIVPPNLILFLRPN
FG05607.1 	HRVFPFARGLFEDKVANFWCAANVVIKLRQWPTELLQRVALGATLASIIPPNAILFLRPR
AN4864.1  	HRIFPFSRGLFEDKVANAWCAIHTFYKLHHFEPELLKRVSLGATLASILIPCAIVFRHPR
          	**:***:********** *** :.. **::: . **:: :* ****:*: *  *:* :*.

MG09310.1 	K--ALLPSAFAATAWGFFLFSYQVHEKSVLLPLMPMTIMLAGSNGLAREVRAWVGFANIL
NCU03317.1	KHPGLLPLAFATTAWGFFLFSYQVHEKSVLLPLMPMTLLLAGRQGLGKDTRAWVGFANIL
FG05607.1 	K--TTLPLAFAATAWGFFLFSYQVHEKSVLLPLMPMTLLLAGKQGLNKDTRAWVGFANLL
AN4864.1  	A--SILLPAFATVGWGFFLFSFQVHEKSVLLPLLPMTLLIAGDGGLNKDTRSWVGWANIL
          	     *  ***:..*******:***********:***:::**  ** ::.*:***:**:*

MG09310.1 	GAWTMFPLLQRVDLRVPYVVLTLLWAYLLGLPPTSTSAYFDDE-----GPATAGQWILFL
NCU03317.1	GCWTMYPLLQRVDLRVPYTVLTLLWAYLLGLPPTSLNAYFQD------GSSIWSQWATAF
FG05607.1 	GVWTMFPLLKRVDLKIPYAVLSLLWSYLLGLPPTSLSAYFQE------GQAAWVQWGTAL
AN4864.1  	GSWTLYPLLKRDGLQVPYFVVTCLWAYLLGLPPTSWQIYRHQRPVGEVEADTEPHGLTRL
          	* **::***:* .*::** *:: **:********* . * .: . ..       :    :

MG09310.1 	VHGAFYLAMGAWHVVDRFFVPPINMPDLWVVANVGLGAVGFALCYFWCTWKLLVDADLIP
NCU03317.1	IHGFFYLTMAFWHVLDMFVVAPKDKPDLWVVANVGVGAAGFSLCYLWCLGSLVIESEILP
FG05607.1 	LHWAFYLAMGAWHALDAFAAPPADKPDLWVVANVGIGAAGFIICYLWCFFKLLQESDLLP
AN4864.1  	IHILFYLAMVGWHVLEAFIPPPPGKPDLWVVLNVLIGAGGFGISYLWCLWKLISLSRRID
          	:*  ***:*  **.:: *  .* . ****** ** :** ** :.*:**  .*:  :  : 

MG09310.1 	KSWYNTKAKVKKG-
NCU03317.1	PSWTGKEAKTKSE-
FG05607.1 	GGSSRSSGKKNKTL
AN4864.1  	SKVEDARKKNQ---
          	        * :
```
